# Influence of internal fixation systems on radiation therapy for spinal tumor

**DOI:** 10.1120/jacmp.v16i4.5450

**Published:** 2015-07-08

**Authors:** Jingfeng Li, Lei Yan, Jianping Wang, Lin Cai, Dongcai Hu

**Affiliations:** ^1^ Department of Orthopedics Zhongnan Hospital of Wuhan University Wuhan Hubei China

**Keywords:** internal fixation system, spinal tumor, treatment planning system, radiation dose

## Abstract

In this study, the influence of internal fixation systems on radiation therapy for spinal tumor was investigated in order to derive a theoretical basis for adjustment of radiation dose for patients with spinal tumor and internal fixation. Based on a common method of internal fixation after resection of spinal tumor, different models of spinal internal fixation were constructed using the lumbar vertebra of fresh domestic pigs and titanium alloy as the internal fixation system. Variations in radiation dose in the vertebral body and partial spinal cord in different types of internal fixation were studied under the same radiation condition (6 MV and 600 mGy) in different fixation models and compared with those irradiated based on the treatment planning system (TPS). Our results showed that spinal internal fixation materials have great impact on the radiation dose absorbed by spinal tumors. Under the same radiation condition, the influence of anterior internal fixation material or combined anterior and posterior approach on radiation dose at the anterior border of the vertebral body was the greatest. Regardless of the kinds of internal fixation method employed, radiation dose at the anterior border of the vertebral body was significantly different from that at other positions. Notably, the influence of posterior internal fixation material on the anterior wall of the vertebral canal was the greatest. X‐ray attenuation and scattering should be taken into consideration for most patients with bone metastasis that receive fixation of metal implants. Further evaluation should then be conducted with modified TPS in order to minimize the potentially harmful effects of inappropriate radiation dose.

PACS number: 87.55.D‐

## I. INTRODUCTION

Spinal tumors can be divided into primary and metastatic types, of which the former accounts for 30% of spinal tumors and 0.4% of all tumors, and of which the latter accounts for 10% to 30% of the new tumors diagnoses annually.^(1)^ Metastatic spinal tumor is the most common of all tumors and can be secondary to any malignant tumor. Spinal metastasis is found in 90% of cancer patients receiving pathological autopsy, and 60% of these metastases are from lung cancer, breast cancer, and prostatic cancer.^2^, ^3^, ^4^ The main therapeutic objectives in both primary and metastatic spinal tumors are to alleviate pain, to maintain or improve neural function, and to maintain and reconstruct spinal stability. Treatment methods include surgical removal of tumor, radiation treatment, and chemotherapy.^(5)^

Although surgical treatment of spinal tumor cannot significantly prolong the life of patients, it can considerably improve their life quality, including delaying the loss of walking ability, eliminating or alleviating pains, and retarding or avoiding paraplegia. Routine methods for internal fixation of spine include anterior internal fixation, posterior internal fixation, and the combination of the two.^(6)^ A variety of materials are used for internal fixation of spine such as stainless steel, titanium alloy, bone cement, and autogenous bone.^(7)^ Currently, the most commonly used internal fixation system consists of a screw‐plate system, nail‐stick system, and titanium mesh.

Although surgical treatment is often sought to remove lesions of spinal tumors, they cannot be completely resected in many cases. Such lesions are treated by postoperative radiation therapy or chemotherapy. Radiation therapy, surgical therapy, and chemotherapy are the three major clinical approaches currently in use for tumor treatment.^(8)^ Radiation therapy can directly kill tumor cells, alleviate pain, prevent and control pathological fracture, and reduce the size of tumors, which creates favorable conditions for surgical resection.^(9)^ According to data from the World Health Organization (WHO), about 70% of tumor patients need radiation therapy and the rate of successful treatment is 45%, which includes 22% by surgical therapy, 18% by radiation therapy, and 5% by chemotherapy.^10^, ^11^ Therefore, radiation therapy is one of the major approaches for treatment of tumor.

Use of an internal fixation system in spines is indispensable for stabilization of spinal structure,^(12)^ recovery of the bearing capacity of spine, and protection of spinal function. However, the effective radiation dose can be altered by nonuniformity of the interface between metal and body tissue.^(13)^ Specifically, the metal used for internal fixation can increase the dose absorbed by the interface between the metal and the tissue on the side where the radiation enters, and reduce the dose absorbed by the tissue behind the metal.^14^, ^15^ Allal et al.^(13)^ used ^60^Co as radioactive source to study variation in radiation absorbed by the tissue at the interface with metal implant and found that radiation dose close to the surface of titanium plate was higher by 5%–7%. However, some studies have reported results that are inconsistent with these findings.^16^, ^17^ Moreover, due to the complexity of the anatomical structure of the spine and the interaction with several internal fixation materials, accurate determination of radiation dose in the vertebral canal and on the vertebral body is very difficult.^(18)^ The radiation tolerance dose of spine is 45 Gy, which is 22 to 25 times that of conventional fractionation.^(19)^ The incidence of myelopathy is equal to 0.2% at the radiation level of 50 Gy, 6% at 60 Gy, and 50% at ~ 69 Gy.^(20)^ Excess radiation can directly or indirectly damage spinal neurons and vascular bed, resulting in radioactive spinal cord injury as well as diffuse and tiny damages on the tissue being irradiated. The damage is usually seen morphologically as demyelination and necrosis. For example, if the influence of titanium plate on radiation dose is neglected in patients undergoing radiation therapy for cervical spine tumor, the esophagus in front of the plate may be irradiated excessively, leading to radioactive esophagitis. Moreover, lesions of vertebral tumor behind are blocked by the titanium plate, so the dose of radiation absorbed will be reduced and the therapeutic goal may not be achieved. Therefore, radiation dose should be adjusted in order to avoid unpredictable and unrecoverable consequences for patients with spinal tumors.

Previous research on the influence of implants on radiation therapy focused mainly on the influence of metal mesh stents placed in the esophagus on the dose of radiation therapy,^21^, ^22^ but less on the spine itself. Following a common method of internal fixation after resection of spinal tumor, different models of spinal internal fixation were constructed using the lumbar vertebra of fresh domestic pigs and titanium alloy as the internal fixation system. Variations in radiation dose in the vertebral body and the spinal cord in different types of internal fixation systems were studied under the same radiation condition (6 MV and 600 mGy). Our results provide a theoretical basis for adjustment of radiation dose in patients with spinal tumor after internal fixation.

## II. MATERIALS AND METHODS

### A. Ethics statement

This study was carried out in strict accordance with the recommendations in the Guide for the Care and Use of Laboratory Animals of the National Institutes of Health. The protocol was approved by the Committee on the Ethics of Animal Experiments of Wuhan University. All animals were sacrificed under pentothal sodium and pentobarbital anesthesia, and all efforts were made to minimize suffering.

### B. Sample processing

Lumbar vertebrae of fresh domestic pigs were used to construct different models of spinal internal fixation. The surrounding muscles and soft tissues were removed, and the spinal dura mater was bluntly dissected and extracted together with the spinal cord. Anterior longitudinal ligament, posterior longitudinal ligament, ligamentum flava, and facet joint capsule were kept intact. The left pediculus arcus vertebrae, transverse process, vertebral lamina, and the tissues between them were resected along the upper edge of L2 vertebral body and the lower edge of L3 vertebral body, respectively, so as to expose the vertebral canals L2 and L3. A fenestra was created on the separated bone from the lateral side.

### C. Establishment of models of spinal internal fixation

#### C.1 Model of anterior fixation with a titanium plate

**Figure 1:**
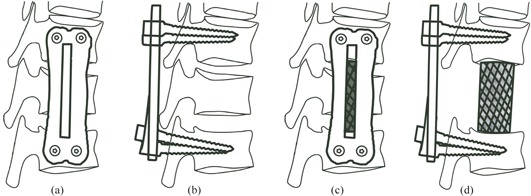
The figures of model of anterior fixation with a titanium plate (a), model of posterior fixation with a nail‐stick system (b), model of fixation by anterior bone grafting/ cement with titanium mesh + anterior screw‐plate (c), and model of fixation by anterior bone grafting/cement with titanium mesh + posterior nail‐stick (d).

The position 1 cm above the lower edge of L1 vertebral body and 1 cm in front of the posterior edge of vertebral body was the entry point of the posterolateral nail from the upper side. The position 1 cm below the upper edge of L3 vertebral body and 1 cm in front of the posterior edge of the vertebral body was the entry point of the posterolateral nail from the lower side. A four‐bore titanium plate (Synthes AG, Bettlach, Switzerland) was fixed on the vertebral bodies L1 and L3. Diagonal drilling, tapping, screwing, and nail fastening were routinely performed (see Fig. 1(a)).

#### C.2 Model of posterior fixation with a nail‐stick system

The isthmus of vertebral laminae L1 and L3 — that is, the junction of the middle line of transverse process and superior articular process — was chosen as the entry point. The bone rongeur was first used to remove the cortical bone covering the entry point, and then the cancellous bone was exposed. A screw was inserted into the anteromedial pediculus arcus vertebrae from the posterior–lateral side using a drilling hammer. The tail of the nail was lifted by 5°–10° and deflected outward by 15°–20°. Pedicle screws (Synthes AG) and connecting rods were inserted after tapping (see Fig. 1(b)).

#### C.3 Models of fixation: anterior bone grafting with titanium mesh + anterior screw‐plate; anterior bone grafting with titanium mesh + posterior nail‐stick

The L3 vertebral body and intervertebral disc were resected using an osteotome and an electric saw, but the posterior wall of the vertebral body and the posterior longitudinal ligament were left intact. The titanium mesh was cut to a height of 36 mm based on the distance between vertebral bodies and volume variation after decompression. The resected bone was cut into pieces, put in the titanium mesh, and compacted. Then the titanium mesh was placed between the vertebral bodies parallel to the anterior border of the vertebral body. Finally, the anterior screw‐plate or the posterior nail‐stick (Synthes AG) was used for fixation, resulting in “anterior bone grafting with titanium mesh + anterior screw‐plate” and “anterior bone grafting with titanium mesh + posterior nail‐stick” fixation, respectively (see Figs. 1(c) and (d)).

#### C.4 Models of fixation: anterior bone cement with titanium mesh + anterior screw‐plate; anterior bone cement with titanium mesh + posterior nail‐stick

The specimens were dissected as described above. A total of 20 g of bone cement (acrylic resin) and 10 ml of water were mixed well and injected into the titanium mesh. After 15 min, the bone cement solidified and the mesh was placed in the position between the vertebral bodies L2 and L4 parallel to the anterior border of the vertebral body. The anterior screw‐plate or the posterior nail‐stick was used for fixation to achieve “anterior bone cement with titanium mesh + anterior screw‐plate” and “anterior bone cement with titanium mesh + posterior nail‐stick” fixation, respectively (see Figs. 1(c) and (d)).

#### C.5 Fixation of a thermoluminescence dosimeter

**Figure 2:**
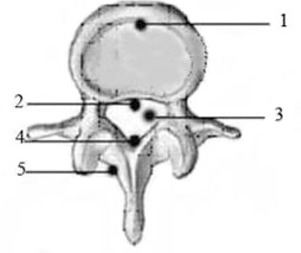
Cross section of the lumbar vertebrae. Numbers show locations of dosimeter placement. 1=anterior border of vertebral body; 2=anterior wall of vertebral canal; 3=center of vertebral canal; 4=posterior wall of vertebral canal; 5=middle part of spinous process.

After preparing the models as described above, five thermoluminescence dosimeters (TLD) were adhered to five reference points on the vertebral canals L2 and L3 with 502 seccotine, and were numbered as shown in Fig. 2. The dosimeters 1 and 5 detect the influence of the internal fixation system on the site of the lesion, while dosimeters 2, 3 and 4 detect its influence on the spinal cord. Next, the bone resected during fenestration was restored and fixed with silk thread.

#### C.6 Establishment of the control group

The control model without any internal fixation material was placed in the water phantom and irradiated using a 6 MV therapy unit. The average radiation dose absorbed by each point was calculated after ten repeated measurements. The correction method is the ETAR method.^(23)^

#### C.7 Group of treatment planning system (TPS)

In order to reduce the influence of metal implants on the dose of radiation therapy as much as possible, a treatment planning system (TPS) (Computerized Medical Systems (CMS)‐XIO, St. Louis, MO), with its pencil beam algorithm, was used for calculation of the isodose curves. TPS was generally employed for simulation calculation so as to correct the radiation dose during radiation treatment planning, which was fixed in energy 6 MV, irradiation dose 600 mGy, radiation field 15×15 cm, source‐to‐tumor distance 108 cm, source‐to‐skin distance 100 cm. In this study, the model of anterior fixation with titanium alloy screw‐plate system, the model of anterior bone cement with titanium mesh + anterior titanium alloy screw‐plate system, the model of anterior bone cement with titanium mesh + posterior nail‐stick system, and the blank control group were compared with the TPS group in order to determine the influence of different internal fixation models on radiation therapy.

### D. Irradiation

The prepared model of spinal internal fixation was placed in water with the spinous process facing upward. Then it was placed on the standard position of X‐ray extraction window of the treatment bed (6 MV treatment unit). The water level was 5 mm above the surface of the spinous process. The center of the L3 vertebral body was 8 cm away from the water surface, and the source‐to‐skin distance was 80 cm. Based on the source‐to‐tumor distance of 88 cm, 60 cGy of radiation dose was selected with a field of 20 cm×20 cm.

Measurements were performed ten times on the specimens of each group. The thermoluminescence elements were taken out and kept still for 48 hr, after which the measurements were read (TLD Reader Model 3000, Kasei Optonix, Ltd., Odawara, Japan) and the averages were calculated.

### E. Statistical analysis

SPSS 20.0 (SPSS Inc., Chicago, IL) was employed to analyze the data and the significance level was set at p<0.05. Comparison between the two models was conducted by the Wilcoxon signed‐rank test. Data for the various reference points in the different models were analyzed by ANOVA, and *t*‐test was conducted to evaluate statistical significance of differences between the two groups.

## III. RESULTS

### A. Fixation models with different internal fixation systems

#### A.1 Model of anterior titanium alloy screw‐plate system, model of anterior bone cement with titanium mesh + anterior titanium alloy screw‐plate system, model of anterior bone cement with titanium mesh + posterior nail‐stick system, and blank control group

No difference was found in radiation doses at the anterior wall, center, and posterior wall of vertebral canal (the inside of vertebral canal). However, doses at the anterior border and the middle part of the spinous process were different from those at other positions.

#### A.2 Model of posterior titanium alloy screw‐plate system

No difference was found among radiation doses of the anterior wall, center, and posterior wall of the vertebral canal. Radiation doses at the anterior wall and the center of the vertebral canal were not different from that in the middle part of the spinous process. However, radiation dose in the posterior part of the vertebral wall was different from that in the middle part of the spinous process (p=0.049). Moreover, the anterior border of the vertebral body had significantly different radiation dose compared with all other positions (1, 2).

**Table 1:** Comparison of radiation doses at each position in different models of internal fixation systems under the same radiation condition

	*Radiation Dose Values (mGy)*				
*Position of TLD*	*Anterior Titanium Alloy Screw‐Plate System* ^a^	*Anterior Bone Cement with Titanium Mesh+Anterior Titanium Alloy Screw‐Plate System* ^a^	*Anterior Bone Cement with Titanium Mesh+Posterior Nail‐Stick System* ^a^	*Posterior Titanium Alloy Screw‐Plate System* ^a^	*Blank Control Group*
1	553.9±8.9	518.9±15.8	550.5±15.5	583.3±29.7	559.9±33.1
2	684.5±28.2	661.7±40.0	692.5±80.5	658.0±18.4	670.7±44.2
3	672.8±34.1	650.9±24.0	666.0±15.0	692.6±40.9	647.1±15.7
4	667.5±28.6	661.3±35.1	693.6±15.7	685.6±21.6	645.7±16.3
5	773.0±34.7	769.5±24.5	781.9±22.3	769.8±34.9	748.8±38.2

a ^a^ Each system model is fixed in energy 6 MV, irradiation dose 600 mGy, radiation field 15×15 cm, source‐to‐tumor distance 108 cm, source‐to‐skin distance 100 cm.

1=anterior border of vertebral body; 2=anterior wall of vertebral canal; 3=center of vertebral canal; 4=posterior wall of vertebral canal; 5=middle part of spinous process.

**Table 2:** Pairwise comparison of radiation doses at different positions in each model of internal fixation system under the same radiation condition

	*P‐values*				
*Position of TLD*	*Anterior Titanium Alloy Screw‐Plate System* ^a^	*Anterior Bone Cement with Titanium Mesh+Anterior Titanium Alloy Screw‐Plate System* ^a^	*Anterior Bone Cement with Titanium Mesh+Posterior Nail‐Stick System* ^a^	*Posterior Titanium Alloy Screw‐Plate System* ^a^	*Blank Control Group*
1 VS 2	0.000	0.000	0.000	0.015	0.001
1 VS 3	0.000	0.001	0.000	0.016	0.002
1 VS 4	0.001	0.000	0.000	0.022	0.002
1 VS 5	0.000	0.000	0.000	0.001	0.000
2 VS 3	0.625	0.834	0.827	0.988	0.620
2 VS 4	0.482	0.866	0.130	0.845	0.577
2 VS 5	0.003	0.002	0.000	0.069	0.002
3 VS 4	0.826	0.706	0.184	0.857	0.949
3 VS 5	0.002	0.001	0.000	0.067	0.001
4 VS 5	0.001	0.002	0.001	0.049	0.001

a ^a^ Each system model is fixed in energy 6 MV, irradiation dose 600 mGy, radiation field 15×15 cm, source‐to‐tumor distance 108 cm, source‐to‐skin distance 100 cm.

1=anterior border of vertebral body; 2=anterior wall of vertebral canal; 3=center of vertebral canal; 4=posterior wall of vertebral canal; 5=middle part of spinous process.

### B. Comparison of treatment groups and TPS group in different internal fixation models

1. Under the same radiation condition, radiation doses at the anterior border of the vertebral body, anterior wall of the vertebral canal, center of the vertebral canal, and middle part of the spinous process of the treatment group increased by 12.26%, 8.08%, 3.60%, and 2.29%, respectively, and the dose on the posterior wall decreased by 1.96% compared with those in the TPS group using the anterior titanium alloy screw‐plate system (Table 3).
2. Under the same radiation condition, radiation doses in the anterior border of the vertebral body, anterior wall of the vertebral canal, center of the vertebral canal, posterior wall of the vertebral canal, and middle part of the spinous process of the treatment group increased by 6.94%, 5.41%, 1.34%, 0.05%, and 5.00%, respectively, compared with those in the TPS group using the anterior bone cement with titanium mesh + anterior titanium alloy screw‐plate system (Table 4).
3. Under the same radiation condition, radiation doses in the anterior border of the vertebral body, anterior wall of the vertebral canal, center of the vertebral canal, posterior wall of the vertebral canal, and middle part of the spinous process of treatment group increased by 8.27%, 7.21%, 5.45%, 7.71%, and 6.51%, respectively, compared with those in the TPS group using the anterior bone cement with titanium mesh + posterior titanium alloy screw‐plate system (Table 5).
4. Under the same radiation condition, radiation doses in the anterior border of the vertebral body, anterior wall of the vertebral canal, center of the vertebral canal, posterior wall of the vertebral canal, and middle part of the spinous process of the treatment group increased by 10.64%, 14.24%, 11.42%, 7.60%, and 10.03%, respectively, compared with those in the TPS group using the posterior titanium alloy screw‐plate system (Table 6).
5. Under the same radiation condition, radiation doses in the anterior border of the vertebral body, anterior wall of the vertebral canal, and middle part of the spinous process of the treatment group increased by 1.68%, 4.32%, and 2.55%, respectively, and the doses in the center and posterior wall of the vertebral canal decreased by 0.80% and 4.54%, respectively, compared with those in the TPS group of the blank controls (Table 7).

**Table 3:** Comparison of treatment group with the anterior titanium alloy screw‐plate system (treatment group) and the TPS group

	*Radiation Doses (mGy)*			*Incremental Percentage (%)*
*Position of TLD*	*Treatment Group* ^a^	*TPS Group* ^a^	*Difference*	
1	553.9	486.0	67.9	12.26
2	684.5	629.2	55.3	8.08
3	672.8	648.6	24.2	3.6
4	667.5	680.6	−13.1	−1.96
5	773.0	755.3	17.7	2.29

a ^a^ Each system model is fixed in energy 6 MV, irradiation dose 600 mGy, radiation field 15×15 cm, source‐to‐tumor distance 108 cm, source‐to‐skin distance 100 cm.

1=anterior border of vertebral body; 2=anterior wall of vertebral canal; 3=center of vertebral canal; 4=posterior wall of vertebral canal; 5=middle part of spinous process.

**Table 4:** Comparison of treatment group receiving anterior bone cement with titanium mesh + anterior titanium alloy screw‐plate system and the TPS group

	*Radiation Doses (mGy)*			*Incremental Percentage (%)*
*Position of TLD*	*Treatment Group* ^a^	*TPS Group* ^a^	*Difference*	
1	518.9	482.9	36.0	6.94
2	656.6	621.1	35.5	5.41
3	650.9	642.2	8.7	1.34
4	661.3	661.0	0.3	0.05
5	769.5	731.0	38.5	5.00

a ^a^ Each system model is fixed in energy 6 MV, irradiation dose 600 mGy, radiation field 15×15 cm, source‐to‐tumor distance 108 cm, source‐to‐skin distance 100 cm.

1=anterior border of vertebral body; 2=anterior wall of vertebral canal; 3=center of vertebral canal; 4=posterior wall of vertebral canal; 5=middle part of spinous process.

**Table 5:** Comparison of treatment group receiving anterior bone cement with titanium mesh + posterior titanium alloy screw‐plate system and the TPS group

	*Radiation Doses (mGy)*			*Incremental Percentage (%)*
*Position of TLD*	*Treatment Group* ^a^	*TPS Group* ^a^	*Difference*	
1	550.5	505.0	45.5	8.27
2	661.7	614.0	47.7	7.21
3	660.0	629.7	36.3	5.45
4	693.6	640.1	53.5	7.71
5	781.9	731.0	50.9	6.51

a ^a^ Each system model is fixed in energy 6 MV, irradiation dose 600 mGy, radiation field 15×15 cm, source‐to‐tumor distance 108 cm, source‐to‐skin distance 100 cm.

1=anterior border of vertebral body; 2=anterior wall of vertebral canal; 3=center of vertebral canal; 4=posterior wall of vertebral canal; 5=middle part of spinous process.

**Table 6:** Comparison of treatment group with the posterior screw‐plate system and the TPS group

	*Radiation Doses (mGy)*			*Incremental Percentage (%)*
*Position of TLD*	*Treatment Group* ^a^	*TPS Group* ^a^	*Difference*	
1	583.5	521.4	62.1	10.64
2	692.5	593.9	98.6	14.24
3	692.6	613.5	79.1	11.42
4	685.6	633.5	52.1	7.60
5	769.8	692.6	77.2	10.03

a ^a^ Each system model is fixed in energy 6 MV, irradiation dose 600 mGy, radiation field 15×15 cm, source‐to‐tumor distance 108 cm, source‐to‐skin distance 100 cm.

1=anterior border of vertebral body; 2=anterior wall of vertebral canal; 3=center of vertebral canal; 4=posterior wall of vertebral canal; 5=middle part of spinous process.

**Table 7:** Comparison of the blank control group with the TPS group

	*Radiation Doses (mGy)*			*Incremental Percentage (%)*
*Position of TLD*	*Treatment Group* ^a^	*TPS Group* ^a^	*Difference*	
1	559.9	550.5	9.4	1.68
2	658.0	629.6	28.4	4.32
3	647.1	652.3	−5.2	−0.80
4	645.7	675.0	−29.3	−4.54
5	748.8	729.7	19.1	2.55

a ^a^ Each system model is fixed in energy 6 MV, irradiation dose 600 mGy, radiation field 15×15 cm, source‐to‐tumor distance 108 cm, source‐to‐skin distance 100 cm.

1=anterior border of vertebral body; 2=anterior wall of vertebral canal; 3=center of vertebral canal; 4=posterior wall of vertebral canal; 5=middle part of spinous process.

## IV. DISCUSSION

Our experimental results show that spinal internal fixation materials have great impact on the radiation dose absorbed by spinal tumors. Under the same radiation condition, the influence of anterior internal fixation material or combined anterior and posterior approach on radiation dose was the greatest at the anterior border of the vertebral body. Irrespective of the internal fixation method employed, radiation dose at the anterior border of the vertebral body was different from that at other positions. Notably, the influence of posterior internal fixation material on the anterior wall of the vertebral canal was the greatest. Taken together, our results show that radiation therapy is influenced by spinal internal fixation materials as follows: 1) X‐ray is attenuated greatly after passing through the spinal internal fixation material, which will affect the radiation dose actually absorbed by the tumor; 2) backscattering of X‐ray by spinal internal fixation materials can result in increased effective radiation dose on the surface of the incident plane; 3) scattered photons and secondary electrons in front of the incident plane of the spinal internal fixation material can enter only partially or cannot enter the emergent plane due to attenuation.^24^, ^25^

Previous studies have shown that spinal internal fixation materials have some effect on radiation therapy of patients with spinal tumors but no obvious impact on chemotherapy.^(26)^ The volume and thickness of spinal internal fixation materials are large in relation to spinal tumors. In cases where the materials are located on the beam path, attenuation of X‐rays is obvious, and increment of radiation caused by scattering will decrease quickly after leaving the interface. Therefore, in addition to attenuation of X‐rays, the influence of scattered rays should also be considered. Moreover, radiation dose for patients with implantation of spinal internal fixation materials should be corrected so as to reduce the probability of failure of radiation therapy and the incidence of side effects.

The determination of radiation dose depends on the specifics of an individual's clinical condition. Although some scholars advocate adjustment of the radiation dose,^(27)^ most advocate irradiation from two fields or irradiation with the same center from several fields instead. The latter methods allow variation of dose to be reduced to 4%, which meets the requirement (Report ICRU 24) that the total uncertainty of dose in the target region must be less than 5%.^21^, ^28^, ^29^ In order to reduce the influence of metal implants on the dose of radiation therapy, the treatment planning system (TPS) is usually employed for simulation calculation.^(30)^ Thus, the radiation dose can be corrected during radiation treatment planning. The calculation of dose with TPS mainly depends on relative electron density, which is derived from CT value. A large artifact may occur when scanning a metal implant with high density, which will result in error in CT calculation. Therefore, the TPS must be modified to take such scenarios into account. Currently, the common correction methods for TPS are the EPL, Batho, and ETAR methods.^23^, ^31^, ^32^ The EPL method is not used widely due to its excessive simplification of the model.^(31)^ The Batho method, or the TAR (tissue‐to‐air ratio) method, consists of 1D nonuniformity correction of microstructures, and is mainly used to correct the influence of nonuniformity information on the major photon transmission path and the distribution of radiation dose.^(23)^ On the other hand, the ETAR method consists of 3D nonuniformity correction of microstructures. In this method, the influence of nonuniform microstructure surrounding the site being irradiated on the distribution of radiation dose is considered on a 3D scale. Since correction by the ETAR method is more effective, it is widely applied in TPS. However, the FDS team thinks that the ETAR method is not accurate enough for calculation of radiation dose, especially for inverse optimization.^(33)^ And the three more sophisticated dose calculation algorithms for TPS in order of increasing accuracy/decreasing performance are pencil beam, superposition/convolution (S/C), and Monte Carlo (MC).^(34)^ In clinical, Monte Carlo method is the unique method able to calculate the dose accurately near a high‐Z inhomogeneity.^(35)^ In this article, models of the anterior titanium alloy screw‐plate system, the anterior bone cement with titanium mesh + anterior titanium alloy screw‐plate system, the anterior bone cement with titanium mesh + posterior nail‐stick system, and the blank control group were compared with the TPS group. Our results showed that spinal internal fixation materials have significant impact on radiation dose, and which could be more accurate corrected by the ETAR method.

The specific method used in clinical research is as follows:^(32)^ if there are no important organs and fast‐reacting tissues near the metal implants (2–3 cm) and the implant is made up of a low‐density metal, modified TPS is used to evaluate the influence of implant on dose distribution. In addition, the influence of X‐ray scattered by the metal in a CT image is also considered in this system. X‐ray attenuation and scattering is considered for most patients with bone metastasis after fixation of metal implants. Further evaluation is then conducted with modified TPS so as to avoid or reduce the effect of incorrect radiation dose.

The results of our study indicate that metal implants have a negative impact on radiation therapy conducted after spinal tumor surgery. However, there is still controversy regarding the best method to determine correct radiation dose. Future studies should focus on ways to avoid the side effects of metal implants, as well as the kinds of metal implants to be used in radiation therapy for different types of bone metastasis.

## V. CONCLUSIONS

X‐ray attenuation and scattering by metal implants should be taken into consideration for most patients with bone metastasis receiving fixation of metal implants. Further evaluation should be conducted with modified TPS so as to avoid or reduce the potentially damaging effects of inappropriate radiation dose.

## ACKNOWLEDGMENTS

This work was financially supported by the Natural Science Foundation of Hubei Province (2010), the National Natural Sciences Foundation of China (No: 81301538), and the Youth Science and Technology Morning Program of Wuhan (Grant No: 2014072704011256).
